# 2D Materials Nanoarchitectonics for 3D Structures/Functions

**DOI:** 10.3390/ma17040936

**Published:** 2024-02-17

**Authors:** Katsuhiko Ariga

**Affiliations:** 1 Research Center for Materials Nanoarchitectonics (MANA), National Institute for Materials Science (NIMS), 1-1 Namiki, Tsukuba 305-0044, Ibaraki, Japan; ariga.katsuhiko@nims.go.jp; 2 Graduate School of Frontier Sciences, The University of Tokyo, 5-1-5 Kashiwanoha, Kashiwa 277-8561, Chiba, Japan

**Keywords:** covalent organic framework (COF), liquid crystal, living cell, metal–organic framework (MOF), nanoarchitectonics, surface, three dimensions, two dimensions

## Abstract

It has become clear that superior material functions are derived from precisely controlled nanostructures. This has been greatly accelerated by the development of nanotechnology. The next step is to assemble materials with knowledge of their nano-level structures. This task is assigned to the post-nanotechnology concept of nanoarchitectonics. However, nanoarchitectonics, which creates intricate three-dimensional functional structures, is not always easy. Two-dimensional nanoarchitectonics based on reactions and arrangements at the surface may be an easier target to tackle. A better methodology would be to define a two-dimensional structure and then develop it into a three-dimensional structure and function. According to these backgrounds, this review paper is organized as follows. The introduction is followed by a summary of the three issues; (i) 2D to 3D dynamic structure control: liquid crystal commanded by the surface, (ii) 2D to 3D rational construction: a metal–organic framework (MOF) and a covalent organic framework (COF); (iii) 2D to 3D functional amplification: cells regulated by the surface. In addition, this review summarizes the important aspects of the ultimate three-dimensional nanoarchitectonics as a perspective. The goal of this paper is to establish an integrated concept of functional material creation by reconsidering various reported cases from the viewpoint of nanoarchitectonics, where nanoarchitectonics can be regarded as a method for everything in materials science.

## 1. Introduction

**Figure 1:**
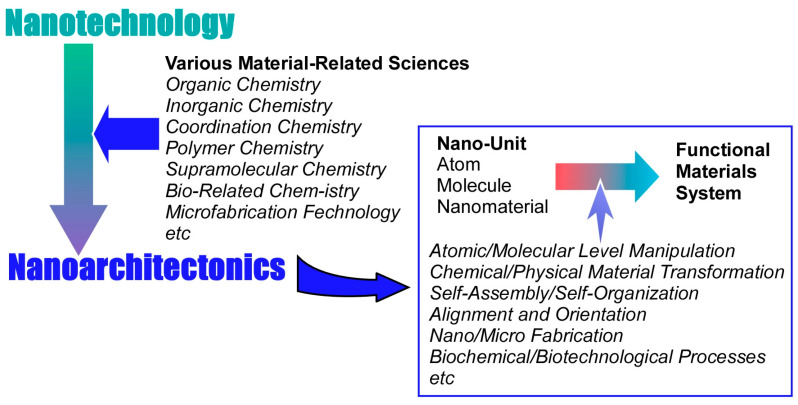
Outline of the nanoarchitectonics concept which establishes a methodology for building functional material systems from nano-units such as atoms, molecules, and nanomaterials.

The development of new technologies such as information technology, device technology, and medical technology is supporting the development of society. However, materials chemistry, which has been steadily developing since the 20th century, is also indispensable [1,2]. Useful materials are essential to fulfill important parts of the later technologies. Such developments in materials chemistry solve a variety of existing problems. Energy production [3,4,5,6,7,8,9,10,11,12,13,14,15,16,17,18], energy storage [19,20,21,22,23,24,25,26,27,28,29,30], environmental remediation [31,32,33,34,35,36,37,38,39,40], carbon neutral strategies [41,42,43,44,45,46], detection of hazardous substances [47,48,49,50,51,52], bio-related sensing [53,54,55,56,57,58], technologies leading to medicine [59,60,61,62,63,64,65,66,67,68,69], and the creation of device materials to support them [70,71,72,73,74,75] are among the many demands that depend on the development of materials chemistry. Effective function is not determined solely by the properties of the substances themselves. Their functions depend much on what kind of structure a substance takes or what kind of internal structure it takes. With the progress of materials chemistry, such matters have been gradually elucidated. This has been greatly accelerated by the development of nanotechnology. As is still the case in current research, nanotechnology makes it possible to observe nanostructures from the atomic and molecular levels [76,77,78,79,80,81,82,83,84,85,86,87,88,89]. Accordingly, it has become clear that superior material functions are derived from precisely controlled nanostructures. The next step is to assemble materials with knowledge of their nano-level structures. This task is assigned to the post-nanotechnology concept of nanoarchitectonics (Figure 1) [90].

Nanotechnology was founded by Richard Feynman in the middle of the 20th century [91,92]. In the early 21st century, nanoarchitectonics was proposed by Masakazu Aono [93,94] as a successor to Feynman’s work. The goal of nanoarchitectonics is to establish a methodology for building functional material systems from nano-units such as atoms, molecules, and nanomaterials [95,96,97]. Rather than a completely new concept, it is more of an integration of existing concepts. In other words, it is a fusion of nanotechnology with various material-related sciences and peripheral fields (organic chemistry, inorganic chemistry, coordination chemistry, polymer chemistry, supramolecular chemistry, bio-related chemistry, microfabrication technology, etc.). The following elemental technologies for building functional material systems from nano-units can be considered: atomic/molecular-level manipulation, chemical/physical material transformation including organic synthesis, self-assembly/self-organization, alignment and orientation by application of external fields and energy, nano/microfabrication, and biochemical/biotechnological processes [98]. These are selected and combined as appropriate. Compared to self-assembly, which is a single-equilibrium process, it is often a multistep process. Accordingly, nanoarchitectonics is well suited to creating asymmetric and hierarchical structures [99]. In addition, the underlying nanoscale interactions often involve uncertainties such as thermal fluctuations, stochastic distributions, and quantum effects. Therefore, the various effects are harmonized rather than simply added together [100].

The above principles are general and Independent of the type of materials and their functions. Nanoarchitectonics will be universally applicable to a wide variety of material systems. Originally, all matter is composed of units of atoms and molecules. Therefore, the concept of nanoarchitectonics, which is the architecture of matter from atoms and molecules, can be the creation of all matter. Like the ultimate theory of everything in physics [101], it could become a method for everything [102,103], an integrated concept for synthesizing functional materials in materials science. In fact, many papers advocating nanoarchitectonics have been published in recent years. The field ranges from basic to applied sciences. It includes material synthesis [104,105,106,107,108,109,110,111,112,113,114], creation of specific structures [115,116,117,118,119,120,121,122,123,124,125], organization of structures [126,127,128,129,130,131,132,133,134,135,136,137,138], exploration of basic physical phenomena [139,140,141,142,143,144,145,146,147,148,149,150], basic biochemistry [151,152,153,154,155,156,157,158,159,160,161,162,163], catalysis [164,165,166,167,168,169,170,171,172,173,174,175], environmental remediation [176,177,178,179,180,181,182,183,184,185,186,187], sensors [188,189,190,191,192,193,194,195,196,197], devices [198,199,200,201,202,203,204,205], energy generation [206,207,208,209,210,211,212,213,214], energy storage [215,216,217,218,219,220,221,222], drug delivery [223,224,225,226,227], cellular control [228,229,230,231], and biomedical applications [232,233,234,235,236,237,238,239,240,241,242]. Since nanoarchitectonics is a comprehensive concept, there are also many approaches that do not advocate nanoarchitectonics but have the same effect as nanoarchitectonics.

However, nanoarchitectonics, which creates intricate three-dimensional functional structures, is not always easy. Two-dimensional nanoarchitectonics based on reactions and arrangements at the surface may be an easier target to tackle. A better methodology would be to define a two-dimensional structure and then develop it into a three-dimensional structure and function. It is a structure development prescription from interface to bulk. Such an approach can be seen in several existing research examples. A typical approach is the interface-based nano thin film fabrication technique. One typical approach is the Langmuir–Blodgett (LB) method [243,244,245,246,247,248,249]. A thin film at the monolayer level is first prepared at a two-dimensional liquid interface, such as a water surface. If these films are sequentially transferred onto a substrate, a three-dimensional, multilayered structure is formed. Layer-by-layer (LbL) assembly is a simpler and more versatile method than the LB method, although its structural control is more ambiguous [250,251,252,253,254,255,256]. Based on specific interactions; thin films are sequentially deposited on a substrate to obtain a variety of thin film structures. If this technique is performed on colloidal templates, it is possible to fabricate three-dimensional thin-film capsules. These techniques are widely studied as a powerful method to convert two-dimensional nanoarchitectonics into three-dimensional structures. However, the resulting structures are still at the thin-film level and may be described as somewhat thicker two-dimensional nanoarchitectonics. Other than these methods, nanoarchitectonics conversion from two dimensional to three dimensional has to be considered.

**Figure 2:**
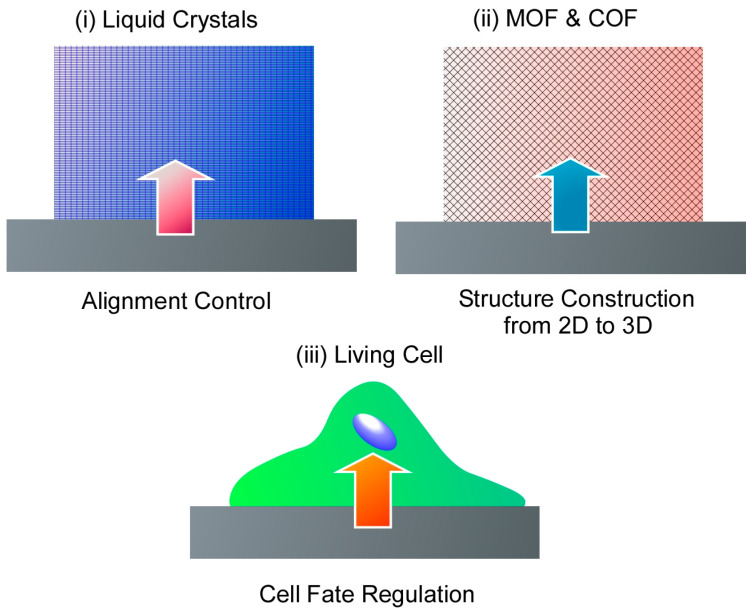
Three topics presented in this review article: (**i**) 2D to 3D dynamic structure control: liquid crystal commanded by the surface; (**ii**) 2D to 3D rational construction: MOF and COF; (**iii**) 2D to 3D functional amplification: cells regulated by the surface.

This review aims to discuss other possibilities of methodologies that expand from some two-dimensional nanoarchitectonics to three-dimensional structures and functions. Three typical examples are presented (Figure 2) that fall under this research orientation, regardless of what is advocated for nanoarchitectonics. The first is the control of bulk materials through nanoarchitectonics of two-dimensional surfaces. This is illustrated by the example of controlling the orientation of a bulk liquid crystal by changing the structure of a very thin surface layer. The second is rational structural architecture from two dimensional to three dimensional. Several examples of metal–organic frameworks (MOFs) and covalent organic frameworks (COFs) from two-dimensional control to three-dimensional architecture are given as examples for this purpose. The third category is the control of cells by surfaces. Cells originally have a cascading signal-generated growth mechanism [257,258,259]. Contact with a two-dimensionally nanoarchitectonized interface can induce advanced functions of the cell. This can be introduced as amplification from two-dimensional nanoarchitectonics to three-dimensional function.

According to the above backgrounds, this review paper is organized as follows. This introduction is followed by a summary of the three issues introduced in the previous paragraphs. They are the following sections: (i) 2D to 3D dynamic structure control: liquid crystal commanded by the surface; (ii) 2D to 3D rational construction: MOF and COF; (iii) 2D to 3D functional amplification: cells regulated by the surface. In addition, this review summarizes the important aspects of the ultimate three-dimensional nanoarchitectonics as a perspective. The goal of this paper is to establish an integrated concept of functional material creation by reconsidering various reported cases from the viewpoint of nanoarchitectonics. Nanoarchitectonics can be regarded as a method for everything in materials science.

## 2. 2D to 3D Dynamic Structure Control: Liquid Crystal Commanded by the Surface

Liquid crystals are materials that can flexibly change their structures while maintaining a certain degree of orientation and other organizing ability [260,261,262]. In Ichimura’s review article on the optical control of liquid crystals [263], the behavior of liquid crystals incorporating photochromic molecules is discussed. Photochromic molecules are usually embedded in various matrices in both fundamental and practical research. In such cases, the structural transformation of the photochromic guest molecule alters the reversible properties of the matrix, the liquid crystal. The orientation of the host molecules and residues that act as the matrix is changed. Broadly speaking, photoaligned liquid crystal systems fall into two types. The first type consists of liquid crystal molecules doped with photochromic units. The light-induced structure of a few photochromic chromophores results in the reorientation of the majority of matrix liquid crystal molecules. This leads to the generation of large optical anisotropy. Another type of liquid crystal system reflects the photoalignment state of photochromic molecules attached to the substrate surface into the bulk liquid crystal layer. This type of structural change in the surface, in which minute structural changes on the surface dictate structural changes in many of the molecular layers above it, is called a command surface [264,265]. This technique demonstrates the effective application of photochromic units to fabricate liquid crystal photoresponsive systems. It also opens the way to the control of photoalignment of liquid crystals, even with irreversible photochemistry. This concept is not only performed by photochromic molecules. It can be a methodology to expand broadly two-dimensional nanoarchitectonics to three-dimensional liquid crystal functionality. It can also be an effective method for fabricating new types of optical elements and devices for photonics applications.

One of the pioneering examples of the command surface concept can be found in the report of Seki, Ichimura, and co-workers [266]. In this study, LB films of side-chain azobenzene amphiphilic polymers were used as photochromic command layers. The photoisomerization of the command layer was used to control the reversible homeotropic planar photochemical orientation of nematic liquid crystals. As a nanoarchitectonics strategy for the command surface, it is important that the azobenzene photochromic unit is separated from the background, the poly(vinyl alcohol) backbone, by a methylene spacer of appropriate length. With this molecular design, a single azobenzene monolayer is sufficient to induce a change in thick liquid crystal orientation. For LB nanoarchitectonics, the vertical transfer method of preparation is more advantageous. The vertical transfer method produced more homogeneously aligned LB films and the liquid crystal molecules were oriented parallel to the immersion direction. When linearly polarized UV light was irradiated, the liquid crystalline molecules reoriented in the direction perpendicular to the polarization plane. In other words, the trans–cis isomerization of the highly photoreactive azobenzene photochromic units in the polymer LB layer successfully commanded the orientation between the homeotropic and planar modes of the nematic liquid crystal.

**Figure 3:**
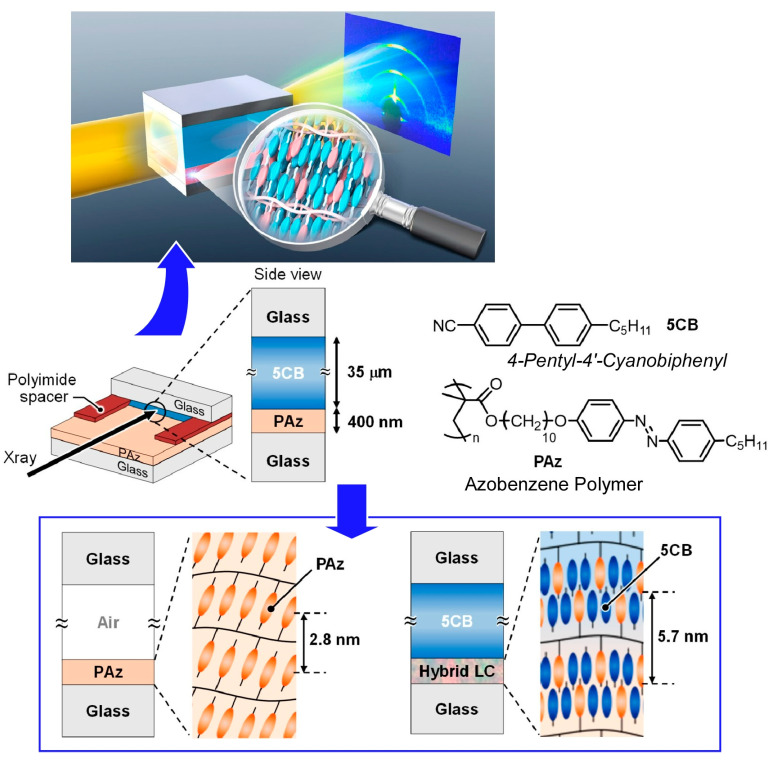
Evaluation of the optical orientation behavior of side-chain liquid crystalline azobenzene polymer films with a thickness of approximately 400 nm and a 35 μm thick low-molecular-weight nematic liquid crystal, 4′-pentyl-4-cyanobiphenyl in situ at the interface. Reprinted with permission from [267]. Copyright 2023 American Chemical Society.

The orientation of liquid crystal molecules is largely governed by the degree of freedom of the surfaces with which they come into contact and other factors. As in the above example, the photoresponsive layer on the surface with freedom serves as the command layer of the side-chain liquid crystal polymer film. Various studies have been conducted to understand the interfacial behavior of these liquid crystal molecules. For example, the photoalignment behavior of nematic liquid crystals on azobenzene polymer films has been the subject of research. Hara, Seki, and co-workers have investigated the optical orientation behavior of side-chain liquid crystalline azobenzene polymer films with a thickness of approximately 400 nm and a 35 μm-thick low-molecular-weight nematic liquid crystal, 4′-pentyl-4-cyanobiphenyl in situ at the interface (Figure 3) [267]. In addition to polarized light optical microscopy observations, small-angle X-ray scattering measurements were used to evaluate the structure inside the liquid crystal-injected sandwich cell. This technique has provided new insights into the behavior of liquid crystal molecules in the vicinity of alignment films on solid substrates. For example, they have succeeded in detecting the selective and time-series structuring and orientation of mesogens at the interface in the formation of smectic layers. A highly ordered smectic liquid crystalline phase is induced by the hybridization of the mesogens of azobenzene polymers and 4′-pentyl-4-cyanobiphenyls. A cooperative hybrid highly ordered smectic liquid crystal phase is formed by weak electron transfer at interfacial contact. Such analysis is important to understand how slight structural changes at the two-dimensional interface are reflected in the three-dimensional structure. Direct X-ray observations in intact liquid crystal cell systems provide useful information on the driving mechanism. It can be a powerful tool in industrial applications in terms of liquid crystal device design. It should also expand the possibilities in terms of practical applications.

**Figure 4:**
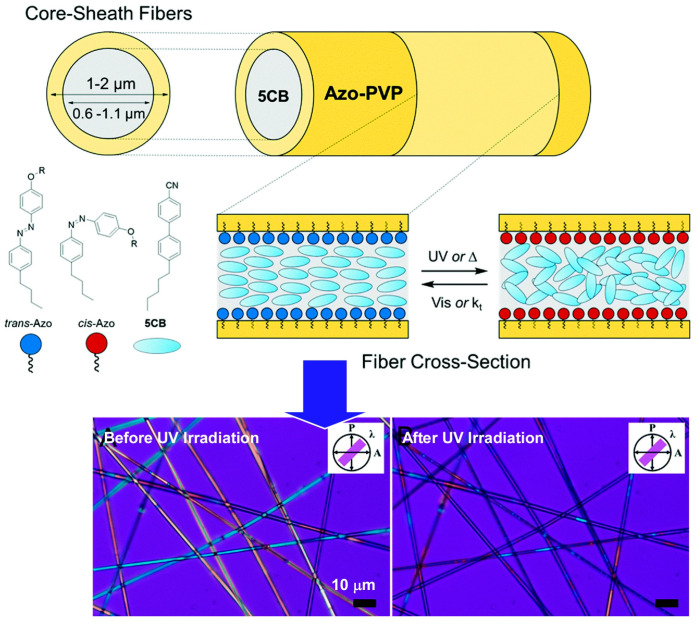
Nematic-to-isotropic transition of liquid crystalline components doped with a photochromic azobenzene surfactant in core–sheath nanofibers. Reprinted with permission from [268]. Copyright 2021 Royal Society of Chemistry.

Lundin and co-workers used electrospinning to fabricate core–sheath nanofibers with photochromic and liquid crystalline components (Figure 4) [268]. The core–sheath nanofibers consist of a polyvinylpyrrolidone sheath doped with a photochromic azobenzene surfactant and a low-molecular-weight nematic liquid crystal core. By incorporating the azobenzene surfactant into the polymer sheath, the nematic-to-isotropic transition temperature of the liquid crystal core could be photochemically controlled. In other words, ultraviolet (UV) irradiation reduced the phase transition temperature. At high surfactant content, the temperature of the photo-induced phase transition was reduced to below room temperature. It allows “turning on” and “turning off” the birefringence of the nanofibers upon UV irradiation and does not require external heating. Photoisomerization of azobenzene surfactants at the poly(vinylpyrrolidone)/liquid crystal interface causes surface-induced disordering of the liquid crystal core. Therefore, UV irradiation causes a change from planar-axis orientation to random orientation. In the case of visible light irradiation, the opposite change is expected to occur. Thus, the photochromic nature of this core–sheath nanofiber system is the result of the difference in the compatibility of the cis- and trans-isomers with the liquid crystal matrix at the liquid crystal/polymer interface. Similar to the command surface of LB films described above, the incorporation of azobenzene into the polymer sheath of liquid-crystalline-core nanofibers allows molecular changes to be reflected in the three-dimensional properties of the liquid crystal.

The optical control of liquid crystal molecules as an example of how two-dimensional nanoarchitectonics through molecular design and orientation is reflected in the properties of three-dimensional materials. Not only LB-type monolayers, but also polymer layers with a core-shell structure act as a command surface. The molecular-level phenomena of structural and orientation changes on the command surface are amplified into three-dimensional material properties such as liquid crystal orientation and phase transitions. The key to this process is molecular phenomena at the interface. The control of the interface leads to the amplification of functional structures from two to three dimensions.

## 3. 2D to 3D Rational Construction: MOF and COF

The synthetic approaches of metal–organic frameworks (MOFs) [269,270,271,272,273,274,275,276,277,278,279] and covalent organic frameworks (COFs) [280,281,282,283,284] are methods for rationally building structures from molecular units and ions. These structures are often formed as two-dimensional structures in interfacial environments, although they can also be obtained in three-dimensional structures such as crystals. As a rational nanoarchitectonics from two dimensions to three dimensions, material designs based on MOFs and COFs are beneficial. In this section, several approaches to three-dimensional architecting from two-dimensional MOFs and COFs are exemplified.

**Figure 5:**
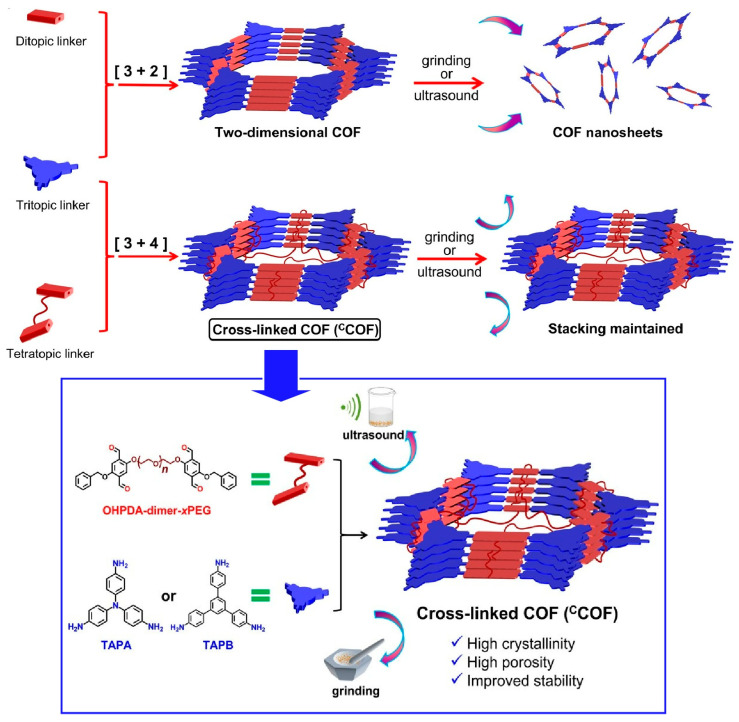
A method to rationally design and synthesize crosslinked COFs in which the two-dimensional COF layers are covalently fixed and linked by poly(ethylene glycol) (PEG) three-dimensional or alkyl chains. Reprinted with permission from [285]. Copyright 2023 American Chemical Society.

Structural evolution from two dimensions to three dimensions is a useful technique for tuning mechanical properties. For example, developing strategies to improve the structural robustness of COFs has been recognized as very important. Yu, Zhang and co-workers reported a method to rationally design and synthesize crosslinked COFs in which the two-dimensional COF layers are covalently fixed and linked by poly(ethylene glycol) (PEG) three-dimensional or alkyl chains (Figure 5) [285]. It is a bottom-up synthetic strategy to structuralize layered structures of two-dimensional COFs using monomers linked by flexible PEG or alkyl chains. The two-dimensional layered structure was converted into a quasi- three-dimensional accumulation framework via covalent crosslinking, where vertical crosslayer bonding is dominant. All synthesized crosslinked COFs were highly crystalline and porous. In particular, they exhibited robust structural stability that surpassed that of typical two-dimensional COFs. While simple assemblies of two-dimensional COFs were easily exfoliated into nanosheets by sonication, pulverization, and water treatment, three-dimensional crosslinked COFs maintained their ordered framework even after such treatments due to the crosslinking effect. The structural stability while inheriting high crystallinity and porosity provides the interlayer stability that is extremely necessary for advanced applications such as heterogeneous catalytic reactions and proton/ion transport. A high application potential of quasi-three-dimensional COFs is expected.

**Figure 6:**
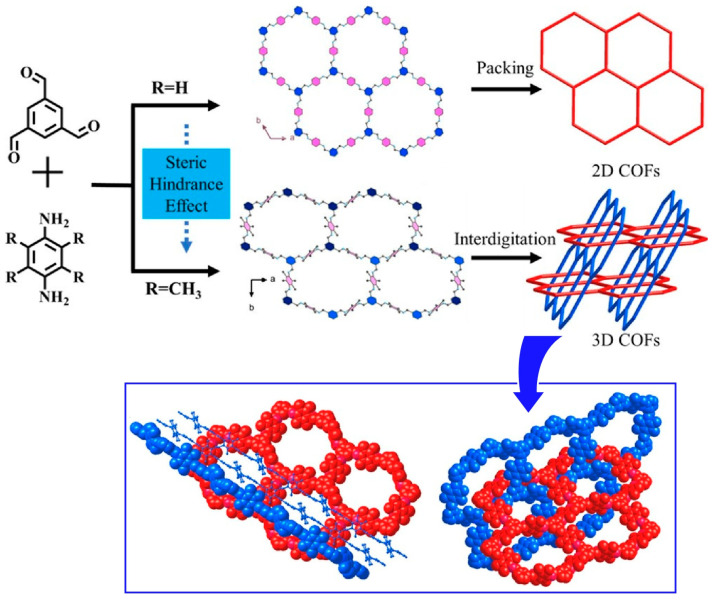
Nanoarchitectonics of three-dimensional COFs based on a two-dimensional network with interpenetrations where the structures were formed by [3+2]imine condensation reactions using triangular knots and linear linkers. Reprinted with permission from [286]. Copyright 2023 American Chemical Society.

Three-dimensional COFs are of interest as a structurally stable group of materials with their inherent large number of open sites and pore confinement effects. However, it is not easy to generate an entangled three-dimensional network formed from multiple two-dimensional layers inclined toward each other. Ma, Li, and co-workers have successfully synthesized a new three-dimensional COF based on a two-dimensional network with interpenetrations (Figure 6) [286]. The structures were formed by [3+2]imine condensation reactions using triangular knots and linear linkers. Specifically, they were formed by [3+2]imine condensation reactions using 1,3,5-triformylbenzene and 2,3,5,6-tetramethyl-1,4-phenylenediamine. The range of strategies for achieving three-dimensional COFs was broadened by mutual coordination. Such examples demonstrate that structurally complex extended frameworks can be obtained from simple molecules and can be used for nanoarchitectonics. They enrich synthetic strategies for three-dimensional COFs and greatly expand the range of COF materials.

**Figure 7:**
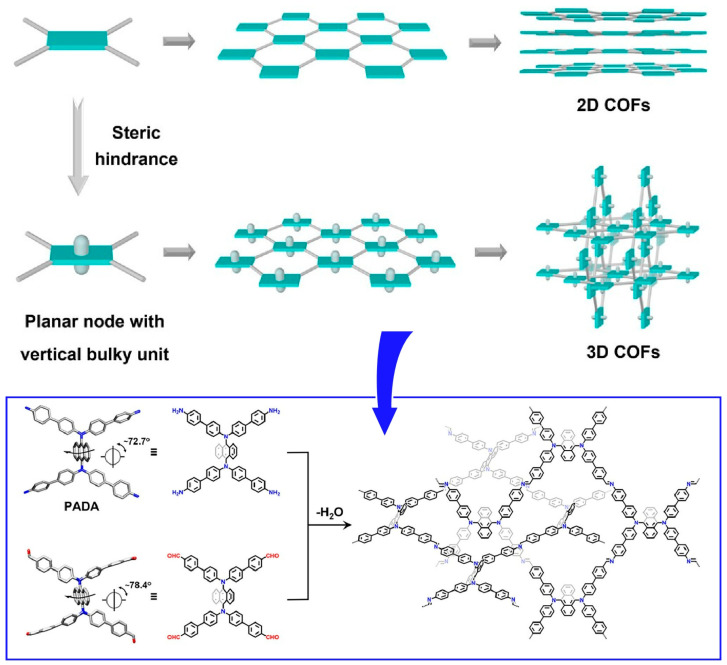
A method to form three-dimensional COFs by introducing steric hindrance to molecular blocks that inhibit π–π stacking, as a method for two-dimensional COFs to intertwine with each other. Reprinted with permission from [287]. Copyright 2023 American Chemical Society.

Gui, Sun, and Wang and co-workers devised a method to form three-dimensional COFs by introducing steric hindrance to molecular blocks that inhibit π–π stacking, as a method for two-dimensional COFs to intertwine with each other (Figure 7) [287]. In this approach, highly crystalline COFs are synthesized starting from rationally designed precursors containing longitudinally bulky anthracene units. Structurally, the presence of anthracene groups outside the C2h symmetry plane strongly inhibits π–π interactions. As a result, the formation of square entanglement is promoted. Furthermore, the fluorescence synthesized here can be used as a sensor to detect trace amounts of antibiotics in water. It is promising to construct three-dimensional COFs by entanglement of two-dimensional layers from precursors with bulky groups in the vertical direction of the skeleton shown in this example. This strategy would open the door for the design and synthesis of many entangled COFs.

**Figure 8:**
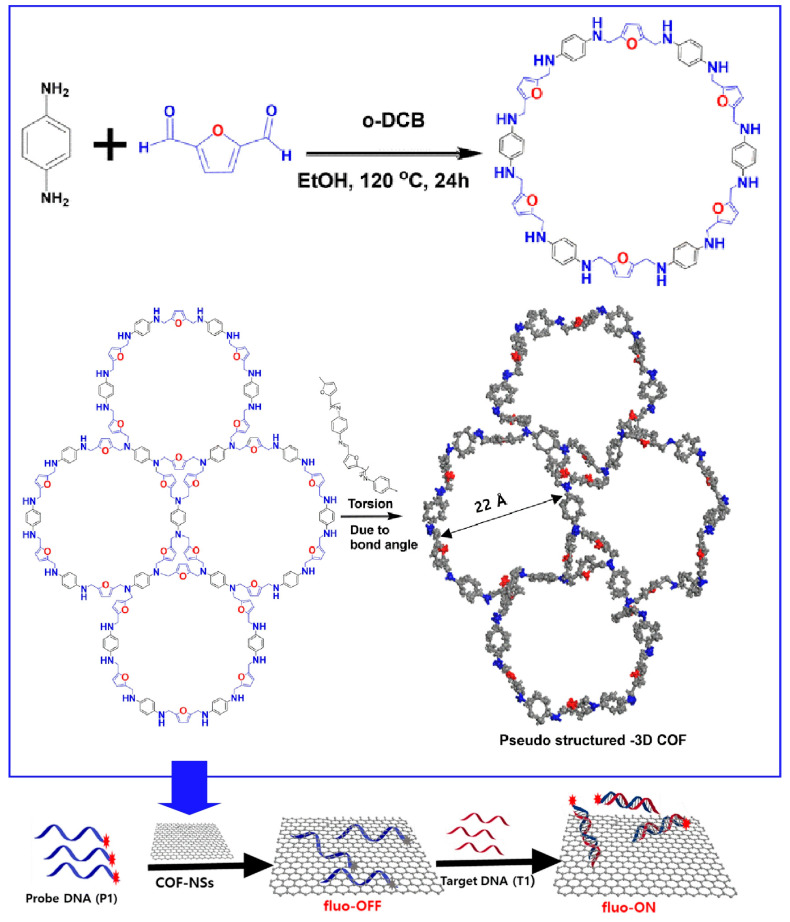
Pseudo-three-dimensional COF nanosheets for detection of biomolecules, including COVID-19 virus where the pseudo-three-dimensional COF nanosheet functions as an adsorbent for biomolecular probes and an acceptor to quench the fluorescence of dye-labeled probes. Reprinted with permission from [288]. Copyright 2023 Royal Society of Chemistry.

COFs can selectively interact with biomolecules due to their large surface area and well-defined pores. Their properties lend themselves to highly sensitive and selective sensing methods. Pseudo-three-dimensional COF nanosheets have shown applications in virus detection, etc. Parvin et al. synthesized pseudo-three-dimensional COF nanosheets by [2+2]imine condensation reaction between building blocks of *p*-phenylenediamine and 2,5-furandicarbonaldehyde [288]. Then, trend-based detection of biomolecules, including COVID-19 virus, was demonstrated (Figure 8). During the synthesis process, a two-dimensional sheet was initially formed, which was later converted into a stable pseudo-three-dimensional structure by changing the bond angles. Furthermore, exfoliation techniques were used to produce nanosheets with reduced π stacking. The exfoliation of COFs into nanosheets provides highly porous structures. The pseudo-three-dimensional COF nanosheet created in this research functions as an adsorbent for biomolecular probes. In addition, it also functions as an acceptor to quench the fluorescence of Texas Red dye-labeled probes. This enabled sensitive and selective fluorescence-based detection of biomolecules, including COVID-19 virus, with a low detection limit of 2 picomoles. Pseudo-three-dimensional COF nanosheets exhibited advantages over conventional graphene oxide, including large surface area, pore structure, specific channel structure, multidimensionality, and stability. Since no catalyst is used in the synthesis process, simpler and more cost-effective production methods can be employed. It also does not require complex and time-consuming procedures such as RT-PCR. These materials have potential applications in the detection of various diseases, electronic devices, and membrane separations.

**Figure 9:**
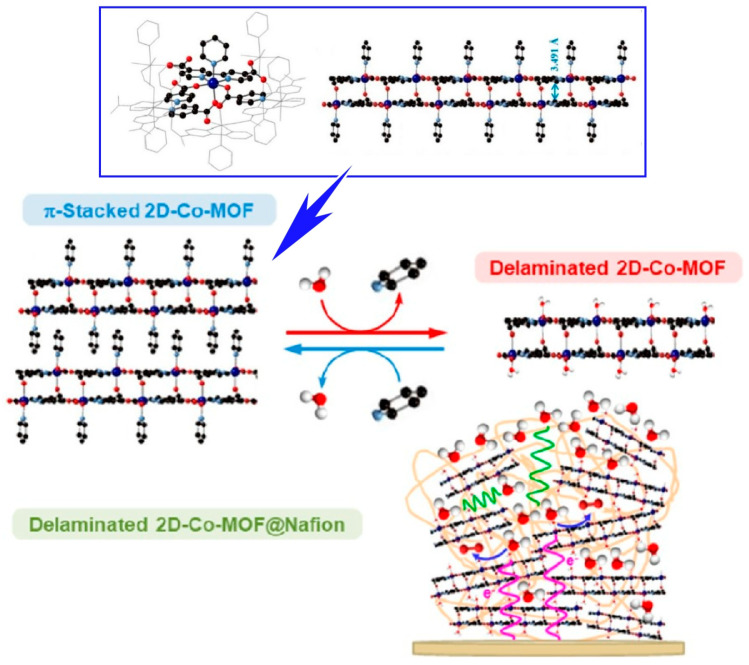
A cobalt MOF based on a well-defined layered double core strongly bound by intermolecular bonds in which its three-dimensional structure is maintained by π–π stacking interactions between the unstable pyridine ligands of the nanosheets. Reprinted with permission from [289]. Copyright 2020 American Chemical Society.

Oña-Burgos and co-workers have developed a stable structure consisting of two pairs of two-dimensional nanosheets [289]. In other words, they synthesized a new cobalt MOF based on a well-defined layered double core strongly bound by intermolecular bonds (Figure 9). Its three-dimensional structure is maintained by π–π stacking interactions between the unstable pyridine ligands of the nanosheets. In an aqueous solution, the axial pyridine ligands are exchanged with water molecules. This results in the exfoliation of the material. In such cases, the individual double nanosheets maintain their structures. Furthermore, the original three-dimensional layered structure is restored by a solvothermal process using pyridine. During the exfoliation-columnarization process, the material exhibits a memory effect. This two-dimensional MOF also exhibits electrocatalytic activity. Electrochemical activation of the two-dimensional cobalt MOF≅nafion-modified electrode improves both ion and electron transfer across the membrane. The formation of electrocatalytically active cobalt centers is then promoted. The activated composite exhibited enhanced electrocatalytic activity for the oxidation of water in neutral media. Spectroscopic and electrochemical characterizations were performed. The nanosheets have a special topology in which the cobalt centers are quite far apart. A mononuclear center-dependent reaction pathway mechanism has been proposed for the cobalt-mediated electrocatalytic oxygen evolution reaction. This electrocatalyst has a better TOF value and robustness than reported for similar electrocatalysts.

**Figure 10:**
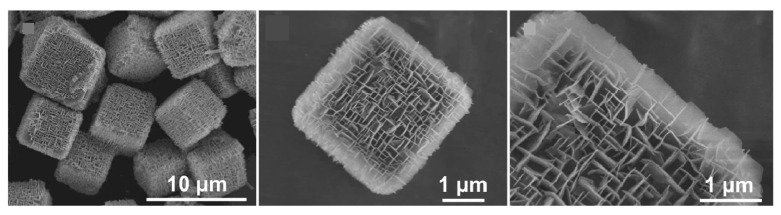
Organometallic frameworks with orthogonal nanosheet arrays for cubic MOFs. Reproduced under terms of the CC-BY license [290]. Copyright 2023 Springer-Nature.

The construction of two-dimensional nanosheets into three-dimensional ordered structures facilitates mass transfer. The full potential of two-dimensional building blocks can be exploited in applications such as catalytic reactions. Zou et al. reported the synthesis of organometallic frameworks with orthogonal nanosheet arrays (Figure 10) [290]. Cubic MOFs are used as the core, and single-crystalline MOF nanosheets with naturally occurring non-preferred faceted exposures are epitaxially grown on top of them as shells. The nanoarchitectonized nanosheets have two typical shapes and crystallographic orientations. Nevertheless, they form an orthogonally aligned single-crystal framework. It is possible to obtain MOFs with a single composition and hollow orthogonally aligned nanosheet morphology. It has the characteristics of peculiar facet exposure and macroporous structure. Therefore, the electrocatalytic oxygen evolution activity is improved compared to conventional nanosheets. This structure exposes unsaturated active sites, stabilizes hydrogen-containing intermediate species, and promotes the oxygen evolution reaction process. The orthogonal arrangement of the nanosheets reduces the possibility of nanosheet re-stacking. Abundant surface active sites are provided, enhancing catalytic activity. Vertical and through pore channels are formed, facilitating diffusion of electrolyte and oxygen molecules. The hollow structure facilitates effective utilization of active sites and mass transfer, improving electrocatalytic properties. Thus, it is expected that rational function-oriented three-dimensional nanoarchitectonics strategies will lead to the design of highly functional heterostructures based on MOF nanosheets.

**Figure 11:**
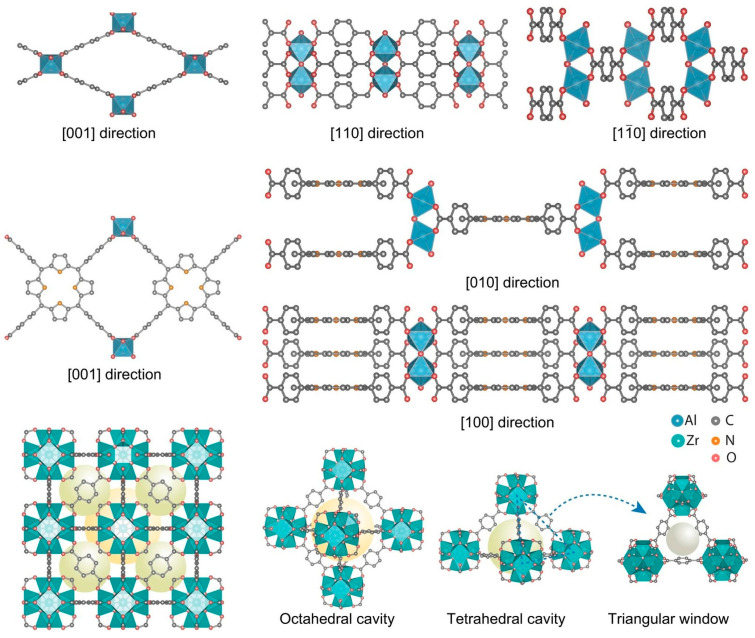
A strategy to grow MOFs into nanochannels with angstrom-scale ion channels with one- to three-dimensional pore structures. Reproduced under terms of the CC-BY license [291]. Copyright 2023 Springer-Nature.

The controllable fabrication of angstrom-sized channels has long been desired in fundamental studies of ion transport. This is also necessary to mimic biological ion channels. Jiang, Zhang, and co-workers reported a strategy to grow MOFs into nanochannels with angstrom-scale ion channels with one- to three-dimensional pore structures (Figure 11) [291]. One-dimensional structures with flexible pore sizes can facilitate cation transport in conductivity and mobility one to two orders of magnitude higher than MOF channels with hybrid pore shapes and sizes. Theoretical simulations and calculations show that the energy barrier for ion transport through pure one-dimensional channels is lower than through complex channel connections. The three-dimensional MOF channel also exhibited better ion sieving properties than the one-dimensional and two-dimensional MOF channels. Further studies assuming angstrom porous MOFs with various channel configurations as the building blocks will open the way to fabricate artificial ion channels of 1 nm or smaller. Applications to high-efficiency ion separation and energy conversion technologies are expected. It will also provide guidance for the development of ion separation and nanofluidics.

**Figure 12:**
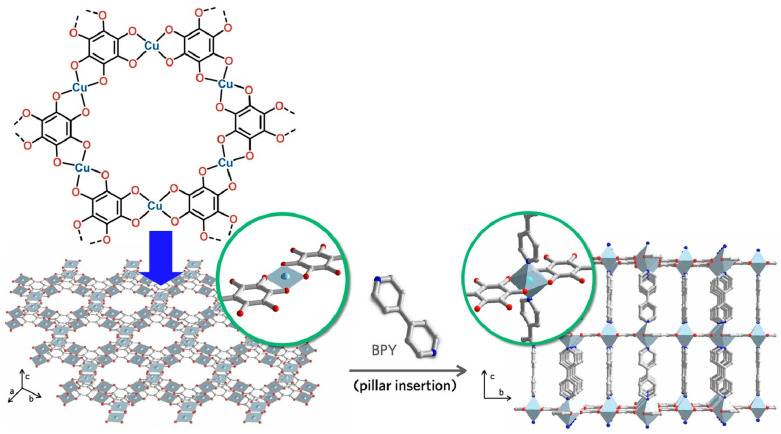
A method for converting two-dimensionally conjugated MOFs into a three-dimensional framework by post-synthetic pillar ligand insertion. Reprinted with permission from [292]. Copyright 2022 American Chemical Society.

Two-dimensional conductive MOFs have attracted much attention for their usefulness in applications ranging from electrochemical energy storage to electronic devices. However, the stacked two-dimensional structure limits access to the internal pores. The full potential has not yet been realized. Park and co-workers reported a method for converting two-dimensionally conjugated MOFs into a three-dimensional framework by post-synthetic pillar ligand insertion (Figure 12) [292]. Such structural transformation improves ion accessibility to the internal pores. As a result, there can be up to a 2-fold increase in capacitance per weight. It is expected that such nanoarchitectonics methods can be used to functionalize a variety of two-dimensional conductive MOFs. Increased accessibility through the introduction of pillars increases the potential for sensing, electronics, and energy-related applications. The pillar portion can also be given a role other than that of a spacer. For example, providing a coordination site for the pillar ligand could lead to advanced applications including electrocatalysis and sensing. The restitution of conductive MOFs from two dimensional to three dimensional and the introduction of additional functionality is expected to facilitate the volatilization of possible materials in this new field.

**Figure 13:**
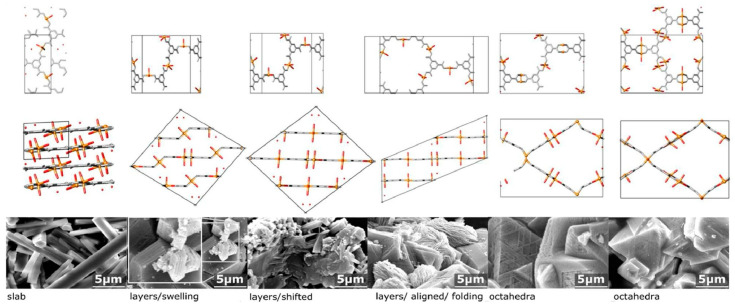
Preparation of a new hierarchical MOF with Cu(II) centers linked by benzene tricarbox-ylates. Reprinted with permission from [293]. Copyright 2017 American Chemical Society.

Wee et al. reported the preparation of a new hierarchical MOF with Cu(II) centers linked by benzene tricarboxylates (BTC) (Figure 13) [293]. It is prepared by thermally induced solid-state transformation of a dense CuBTC precursor phase. It is found that ribbon-like one-dimensional constituent units transform into two-dimensional layers and finally into a three-dimensional network. The formed phases contain excess copper. The charge is compensated by hydroxyl groups, forming an open microporous framework with microporosity. This structure is useful for molecular separation. For example, it was superior to other hierarchical materials in the separation of 11-component mixtures of C_1_–C_6_ alkanes. Microscopic insights into structural host–guest interactions were obtained, confirming a significant entropic contribution to molecular separation.

**Figure 14:**
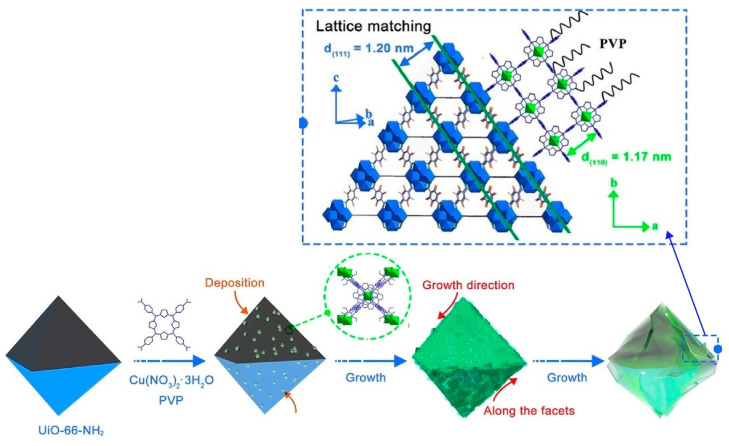
Synthesis of a two-dimension-on-three-dimension (2D-on-3D) hetero-MOF structure. Reprinted with permission from [294]. Copyright 2022 Wiley VCH.

Li and co-workers reported the synthesis of a two-dimension-on-three-dimension (2D-on-3D) hetero-MOF structure (Figure 14) [294]. They introduced a kinetic control method using polyvinylpyrrolidone to realize an anti-epitaxial growth pattern of foreign MOF nuclei on the (111) plane of the UiO-66-NH_2_ octahedron. This nanoarchitectonics methodology has led to the successful fabrication of 2D-on-3D MOFs with various heterostructures (Cu, Zn, Cd, Co, Ni). 2D-on-3D MOFs exhibit unique dimensional hybridization effects in the photocatalytic hydrogen evolution process in a photocatalytic hydrogen evolution process. The photoactivity was significantly enhanced compared to the usual dimensionally identical two-dimensional, three-dimensional, and 3D-on-3D MOF structures. This can be attributed to faster electron transfer rates and more efficient electron–hole separation.

**Figure 15:**
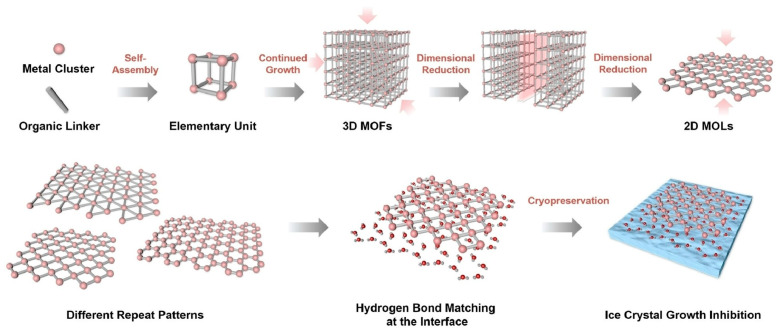
Synthesis of a series of hafnium (Hf)-based two-dimensional metal–organic layers with different thicknesses (from single layer to stacked multilayers) and densities of hydrogen-bonding sites with inhibition capability of ice growth. Reprinted with permission from [295]. Copyright 2023 Wiley VCH.

Conversely, there are examples where dimensional reduction nanoarchitectonics of MOF structures from three dimensions to two dimensions is functionally superior. For example, a dimensional reduction approach has been proposed to improve the cryopreservation efficiency of red blood cells by MOFs. Such an approach takes the methodology of a stepwise reduction in three-dimensional MOF nanoparticles into two-dimensional ultrathin metal–organic layers (MOLs). Guo, Zhu, and co-workers synthesized a series of hafnium (Hf)-based two-dimensional metal–organic layers with different thicknesses (from single layer to stacked multilayers) and densities of hydrogen-bonding sites (Figure 15) [295]. Two-dimensional MOLs enhance the interaction of interfacial hydrogen-bonded water networks due to their high surface-to-volume ratio. This increases the utilization of internally ordered structures. The ability of the hydrogen donor group to recognize and match ice crystal planes can effectively inhibit ice growth and recrystallization for red blood cell cryopreservation. Thin-layered Hf-MOL was found to have significantly better ice recrystallization inhibitory activity and superior cell retrieval efficiency compared to three-dimensional MOL. Flexible two-dimensional MOLs have a higher density of hydrogen donor groups compared to three-dimensional MOF nanoparticles, which have a rigid structure and limited exposure of lattice planes. They also have smaller steric hindrance. These structural advantages may account for the marked efficiency of MOFs in ice suppression and erythrocyte recovery.

MOF and COF nanoarchitectonics approaches are excellent methods for rationally designing and building material structures from unit molecules and ions. Compared to conventional material synthesis methods, MOF and COF have a short history of development and leave plenty of room for various developments. The excellent dimensionality controlled nanoarchitectonics method is expected to contribute to the development of various functional systems.

## 4. 2D to 3D Functional Amplification: Cells Regulated by the Surface

Within living cells, various functional units function through cascade-like coordination. Thus, external stimuli and molecular inputs often lead to sophisticated functions. This can be a useful system for linking two-dimensional nanoarchitectonics to three-dimensional function. Thus, creating artificial structures in two dimensions and altering the function of the cells that come into contact with them can be very sophisticated transfer from two-dimensional to three-dimensional nanoarchitectonics function. This section presents examples of cellular control by two-dimensional liquid interfaces and nanoarchitectonized two-dimensional structures.

**Figure 16:**
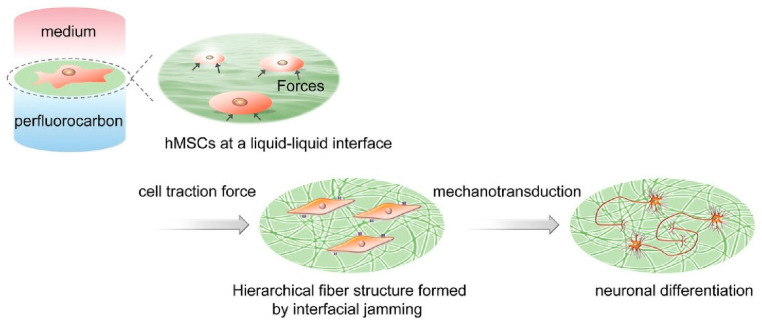
A technique that uses the interface between two immiscible liquids, aqueous cell culture solutions and perfluorocarbons, as a site for culturing and inducing differentiation of human mesenchymal stem cells (hMSCs). Reprinted with permission from [296]. Copyright 2019 Wiley VCH.

Cells are cultured on solid interfaces such as conventional culture media. In the culture process, cells are affected by the solid surface of the equipment, such as mechanical properties. On the other hand, cell control at the liquid interface, which is unaffected by materials such as containers, is a pioneer area. In fact, liquid interfaces are found in many places as adaptive systems in biological systems, from the tear film of the eye to the liquid lining of the lungs and stomach. At such interfaces, liquid interfaces are ubiquitous in natural adaptive systems. The fluidity and reconfigurability of liquids allow for unique response mechanisms. By anchoring living cells at the liquid interface, a system can be constructed that dynamically adapts to the forces generated by the cells. Such cell research at the soft liquid interface is attracting increasing attention. Jia et al. have successfully developed a technique that uses the interface between two immiscible liquids, aqueous cell culture solutions and perfluorocarbons, as a site for culturing and inducing differentiation of human mesenchymal stem cells (hMSCs) (Figure 16) [296]. At this interface, coexisting fibronectin proteins form organized nanosheets that exhibit transient mechanical effects with the cells. Early in culture, the traction force of the cells dynamically transformed the two-dimensional protein nanosheet into a hierarchical fiber structure. Elongated fibronectin aggregates modulate the size and elongation of the focal adhesions of the interacting network. Ultimately, these mechano-transduction signals determine the fate of stem cell differentiation. In this system, cultured hMSCs spontaneously differentiated into neurons without the addition of differentiation-inducing factors. The hMSCs adhered to the liquid interface and took an elongated adherent shape. Stress fibers of F-actin originating there were formed. The accumulation of downstream signaling protein phosphatase (FAK) activators in adhesion plaques and nuclear migration of Yes-associated protein (YAP) were also observed. hMSCs do not perceive protein nanosheets as soft, suggesting that some novel mechano-sensing mechanism is involved. Depending on the design of the two-dimensional nanoarchitectonics, it may be possible to induce differentiation into various types of cells, not only neural cells. This methodology is expected to lead to the development of new technologies for regenerative medicine.

**Figure 17:**
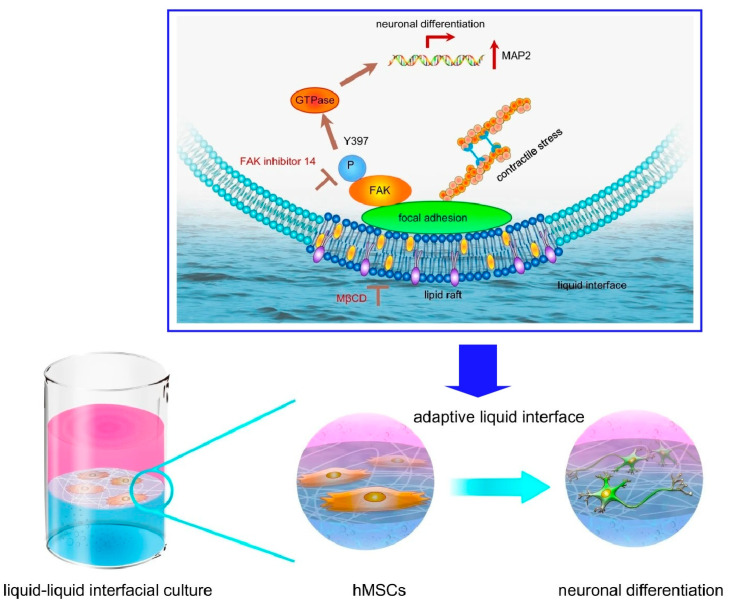
Culture on a two-dimensional network of protein nanofibrils at the liquid–liquid interface for neural differentiation of hMSCs where lipid raft microdomains were found to play a central regulatory role in both the initial cell adhesion and subsequent neural differentiation of hMSCs. Reproduced under terms of the CC-BY license [297]. Copyright 2022 Springer-Nature.

Protein nanofibrils are polymeric β-sheet aggregates of proteins of several microns in length. This structure is also a promising material to mimic extracellular matrix (ECM) matrix fibers due to its biocompatibility for cell adhesion and high mechanical strength. Jia et al. created an adaptive biomaterial based on a two-dimensional network of protein nanofibrils at the liquid–liquid interface and cultured hMSCs [297]. Culture on a two-dimensional network of protein nanofibrils at the liquid–liquid interface promoted neural differentiation of hMSCs. Throughout the study, lipid raft microdomains were found to play a central regulatory role in both the initial cell adhesion and subsequent neural differentiation of hMSCs (Figure 17). They are receptive to biophysical stimuli involving the lipid raft/focal adhesion kinase (FAK) pathway. It seems to direct the neural differentiation of hMSCs. Lipid rafts internalize cell adhesion molecules. In addition, lipid rafts act as an enrichment platform. This induces the integration of large signaling complexes. Through these processes, cells can rapidly adapt to the constantly changing microenvironment. FAK is one of the key mechano-sensors at the adaptive liquid interface. Spatiotemporal regulation of FAK phosphorylation is a prerequisite for neural differentiation of hMSCs. Lipid raft formation and FAK phosphorylation at the adaptive liquid interface regulate hMSC differentiation. These findings also provide a better understanding of the fundamentals of cell-ECM dynamic interactions. Two-dimensional nanoarchitectonics can also incorporate bioactive proteins and responsive polymers. Two-dimensional nanoarchitectonics of liquid interfaces may provide opportunities to design adaptive biomaterials that were previously unimaginable.

**Figure 18:**
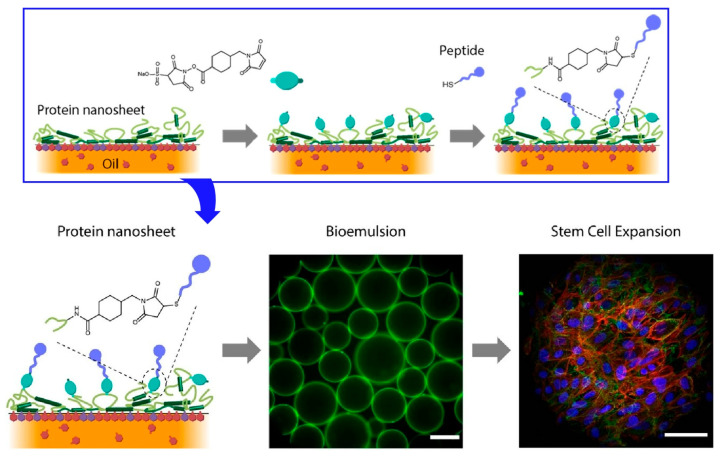
Interfacial dynamics of bovine serum albumin (BSA) and β-lactoglobulin (BLG) aggregates at the fluorinated liquid-water interface where high cell proliferation can be achieved even on bioemulsions with protein nanosheet formation without surfactants. Reproduced under terms of the CC-BY license [298]. Copyright 2023 American Chemical Society.

Gautrot and co-workers examine the interfacial dynamics of bovine serum albumin (BSA) and β-lactoglobulin (BLG) aggregates at the fluorinated liquid-water interface (Figure 18) [298]. The design of protein nanosheets based on these two globular proteins biofunctionalized with RGDSP peptides that enable cell adhesion is mentioned. High cell proliferation can be achieved even on bioemulsions with protein nanosheet formation without surfactants. As a strategy for the rational design of scaffold proteins at liquid interfaces, the fabrication of interfaces with strong shear dynamics and elasticity, bioactivity, and cell adhesion was investigated. For example, in the case of BLG nanosheets, relatively high elasticity was observed even in the absence of the co-surfactant. Based on this as well, the research demonstrated adhesion and proliferation of mesenchymal stem cells and human embryonic kidney (HEK) cells on bioemulsions stabilized with RGD-functionalized protein nanosheets. Such protein nanosheets and bioemulsions are useful for the development of bioreactors for scale-up of cell production. Such studies could also lead to the control of biological activity with scaffold proteins commonly used in food processing and stem cell technology, without the use of surfactant molecules.

**Figure 19:**
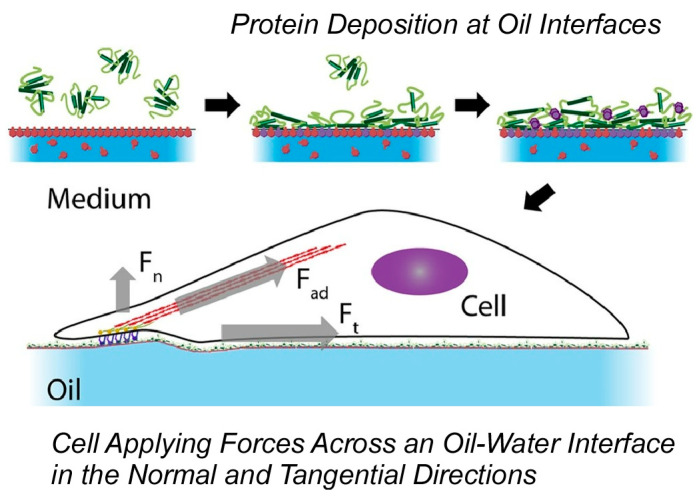
Cell spreading and growth on low-viscous liquid surfaces enabled by the self-assembly of mechanically strong protein nanosheets at the interface. Reproduced under terms of the CC-BY license [299]. Copyright 2018 American Chemical Society.

The use of liquid interfaces for cell culture has the potential to avoid the inconveniences of using solid substrates. In particular, it is advantageous in terms of scale-up and cell detachment. This methodology could be applied to other biotechnological platforms, such as microdroplet systems. For this purpose, analytical studies are needed. Gautrot and co-workers reconfirmed that cell spreading and growth on low-viscous liquid surfaces are enabled by the self-assembly of mechanically strong protein nanosheets at the interface [299]. Interfacial rheology and atomic force microscopy measurements revealed the mechanical properties of protein nanosheets and their associated liquid interfaces. The aggregation behavior of surfactant molecules with proteins and polymers associated at the liquid interface is directly related to the interfacial mechanics. Cells do not rely on surface tension to sustain diffusion, as in the case of amoebae, but primarily sense the in-plane mechanical properties of the interface (Figure 19). Based on these findings, it is essential to design bulk and nanoscale mechanical properties independently. Both scales can provide suitable structures and control cellular phenotypes. In other words, the design of biomaterials and implants requires nanoscale material design, where cell adhesion phenomena can be designed at the interface, independently of other bulk properties needed to provide flexibility and structure.

Gautrot and co-workers showed that mesenchymal and adherent stem cells can be cultured on liquid surfaces [300]. They also exemplified that this is mediated by the association of polymer nanosheets at the liquid–liquid interface. Despite the lack of bulk mechanical properties of the underlying liquid substrate, cell adhesion to the quasi-two-dimensional material is mediated by an integrin/actomyosin mechanism. Stem cell proliferation and differentiation are also controlled by the mechanical properties of the self-assembled protein nanosheets. Keratinocytes spread on rigid poly(l-lysine) nanosheets formed a structured actin cytoskeleton with distinct stress fibers. Focal adhesions were also formed. In contrast, cells adhering to the soft poly(l-lysine)-oil interface did not observe such structures. The use of solid substrates often requires potentially harmful enzymatic degradation for cell recovery. The use of liquid substrates avoids such problems.

**Figure 20:**
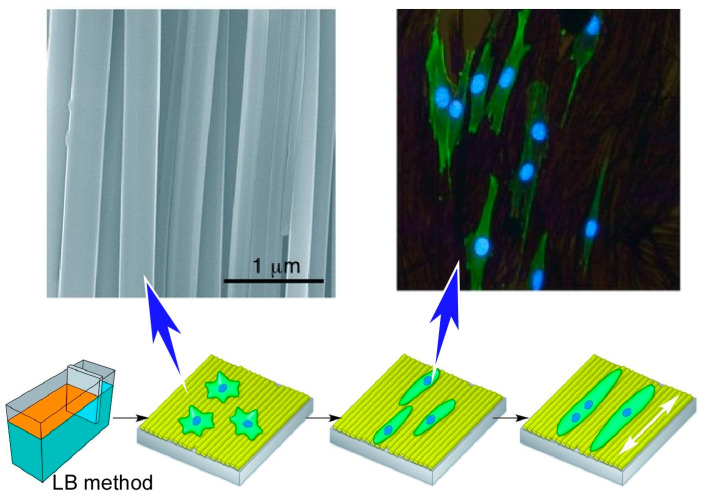
Muscle differentiation and simultaneously controlling the direction of cell growth on the two-dimensional in-plane aligned structure of fullerene nanowhiskers as a cell scaffold. Reprinted with permission from [310]. Copyright 2015 Wiley VCH.

Cells are controlled not only by two-dimensional nanoarchitectonics, such as protein nanosheets that spontaneously form at the liquid interface, but also by the two-dimensional structure of artificially placed nanomaterials. For example, the assembly of fullerenes, which are nanomaterials of carbon materials, is often used in two-dimensional nanoarchitectonics. Although fullerenes are zero-dimensional spherical structures, they exhibit a variety of self-assembled structures from one dimension to three dimensions, such as sheets, rods, pores, and whiskers [301,302,303,304,305,306,307,308,309]. For example, fullerene nanowhiskers can be prepared on a large scale using the liquid–liquid interface deposition method. Minami and co-workers have successfully induced muscle differentiation by arranging fullerene nanowhiskers while simultaneously controlling the direction of cell growth (Figure 20) [310]. The two-dimensional in-plane aligned structure of fullerene nanowhiskers can be applied as a cell scaffold, and the orientation of muscle fibers formed during muscle differentiation can be controlled by the orientation of fullerene nanowhiskers. Using the Langmuir–Blodgett (LB) method, highly aligned one-dimensional fullerene nanowhisker scaffolds were fabricated in the centimeter region. This scaffold can simultaneously control cell orientation and differentiation into muscle cells C2C12 myoblasts. Subsequently, myogenic differentiation and cell growth direction were analyzed by immunostaining for myosin heavy chain. A protein required for nucleus and myotube formation C2C12 myoblasts were found to fuse and form multinucleated myotubes. The fusion index increased from 12.3% on glass to 23.2% on aligned fullerene nanowhisker scaffolds. One-dimensional fullerene nanowhiskers stimulate myoblast fusion. The direction of myotube formation strongly coincided with the direction of aligned fullerene nanowhiskers. When the same experiment was performed on bare glass, myoblasts fused randomly.

**Figure 21:**
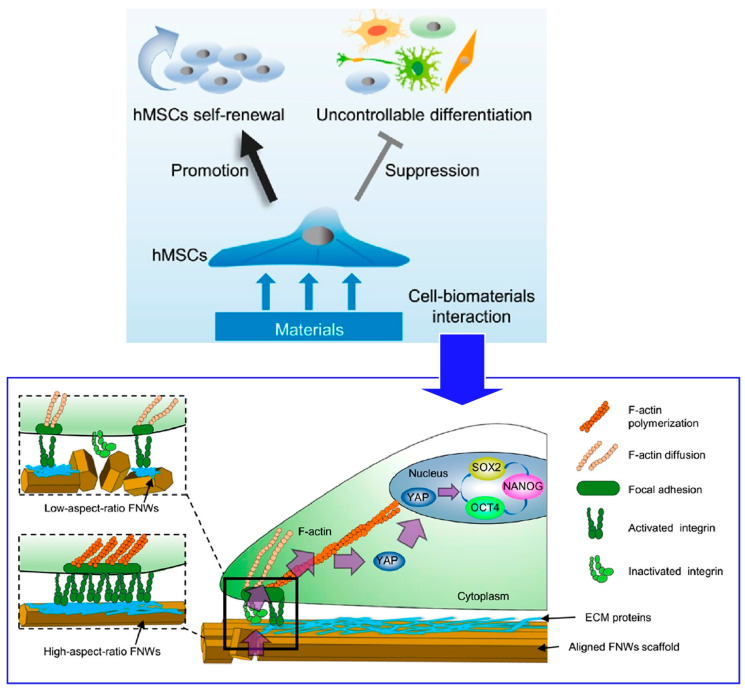
Nanopatterned surfaces fabricated with high-aspect-ratio fullerene nanowhiskers for long-term pluripotency retention and differentiation potential of hMSCs where mechanical signals are transmitted to the nucleus by YAP and YAP translocation to the nucleus positively regulates the activity of core regulators (OCT4, SOX2, NANOG). Reproduced under terms of the CC-BY license [311]. Copyright 2020 American Chemical Society.

hMSCs are useful for cell-based tissue regeneration therapy because of their easy availability and potent immunosuppressive properties. However, the therapeutic efficacy based on hMSCs is limited by the small amount of human-derived cells isolated for clinical use. During in vitro growth, hMSCs undergo uncontrolled differentiation. In the process of in vitro proliferation, hMSCs undergo uncontrolled differentiation, thereby rapidly losing pluripotency and regenerative capacity. Therefore, new strategies to grow hMSCs in vitro while preserving their stem cell properties are strongly needed. This technique can produce large-area nanostructured surfaces with continuously adjustable alignment of the constituents. Nanopatterned surfaces fabricated with high-aspect-ratio fullerene nanowhiskers could promote long-term pluripotency retention and differentiation potential of hMSCs via appropriate cell contractility and nuclear localization of Yes-associated protein (YAP) as demonstrated by Song et al. (Figure 21) [311]. Mechanical signals are transmitted to the nucleus by YAP. As a transcriptional coactivator, YAP translocation to the nucleus positively regulates the activity of core regulators (OCT4, SOX2, NANOG). As a result, retention of pluripotency of hMSCs is promoted. High aspect ratio fullerene nanowhiskers as cell culture scaffolds create an intermediate situation where focal adhesion is effectively reduced but not eliminated. As a result, long-term proliferation of hMSCs that retain pluripotency is mediated by appropriate cell contractility and nuclear localization of YAP. The pseudo-LB method used here is a simple technique that can be manipulated manually. It can be easily used to fabricate centimeter-sized nanotopographic substrates for the mass proliferation of hMSCs in clinical practice. This study demonstrates the importance of two-dimensional nanoarchitectonics for improving hMSCs technology in regenerative therapies.

Cells have well-developed cascade-like information transmission systems, which are excellent systems for generating large functions from simple stimuli. Exposing cells to structure-controlled two-dimensional surfaces allows for the expression of sophisticated functions such as cell differentiation and proliferation. Various scaffolds such as protein nanosheets and very rigid fullerene nanowhisker arrays, which form in very soft fields such as liquid surfaces, produce diverse cellular changes. This is not only of basic scientific interest as two-dimensional nanoarchitectonics makes a breakthrough into three-dimensional function. This is an area that should also make a significant contribution to practical areas such as regenerative medicine.

## 5. Summary and Perspectives

Nanoarchitectonics is a post-nanotechnology concept that provides functional material systems from nano-units (atoms, molecules, and nanomaterials). Interfacial media contribute sufficiently to building functional materials. This is where two-dimensional (2D) materials as building units of three-dimensional (3D) functional nanoarchitectures are powerful. On top of that, materials nanoarchitectonics from two dimensions to three dimensions is necessary for the development of a wider range of functional materials. In this review, the control of liquid crystals from two dimensions, three-dimensional architecture of MOF/COF structures, and the control of cell arrangement and differentiation by two-dimensional contact are taken as examples.

Molecular films made of LB-type monolayers can control the orientation of liquid crystals as a command surface. The molecular-level phenomena of structural and orientation changes on the command surface are amplified into three-dimensional material properties such as liquid crystal orientation and phase transitions. This indicates that the combination of a soft oriented structure and a precise surface is useful. MOF and COF are methods that enable rational design and construction of material structures from unit molecules and ions. The structure produced is determined by the arrangement of interacting functional groups in the molecular unit and the coordination structure of the ions. This means that the two-dimensional and three-dimensional structures of MOFs and COFs are determined by the structural design of the molecular unit-ion unit in the zero dimension. Therefore, the conversion from two dimensional to three dimensional can be achieved by devising the combination of the two-dimensional and three-dimensional structures. For structures with fixed interaction points, the structural design of the units is shown to be useful. Organisms are complex complexes of functions. However, cells have well-developed cascade-like information transmission systems. By exposing cells to structure-controlled two-dimensional surfaces, sophisticated functions such as cell differentiation and proliferation are expressed. This is a rational system for generating large three-dimensional functions from simple stimuli from two dimensions. Utilizing the cascade stimulus transduction system of living organisms is also useful for generating three-dimensional functions from two-dimensional nanoarchitectonics. In addition to approaches and examples described in this review article, simulations/theoretical perspective of 2D materials nanoarchitectonics for 3D functionalities are crucial matters. For example, both DFT calculation [312] and ab initio molecular dynamics [313] are rapidly transforming the field. With the aids of experimental and theoretical considerations, representative differences between 2D and 3D materials will be more clearly elucidated. Such additional efforts would lead to more sophisticated transformation from 2D to 3D from viewviewpoints of applications.

The several examples presented here have extracted several keys to increasing two-dimensional nanoarchitectonics to three-dimensional functionality. These include the use of soft orientation systems, the use of structural architecture reflecting molecular unit structures, and the use of cascade-like function transfer systems in living organisms. These are typical examples, but there may be other ways to link two-dimensional nanoarchitectonics to three-dimensional functionality. The ultimate goal of nanoarchitectonics is to create bio-like functional materials whose functions are hierarchical and exhibit complex interactions. Therefore, we must consider systems that incorporate multiple and diverse multifunctional mechanisms in three-dimension as described above. There are an infinite number of choices of substances, actions, and functions, and their combinations are also diverse. Therefore, there may be a limit to the trial-and-error or few-theory-based approach. Fortunately, humankind has developed artificial intelligence technology. In the field of materials science, machine learning approaches [314,315,316] and the concept of materials informatics [317,318,319] are being used. The combination of nanoarchitectonics and materials informatics has also been proposed [320,321]. The fabrication of functional structures from a large number of options may be completed with the help of artificial intelligence under the concept of nanoarchitectonics. Finally, these developments are expected to become nanoarchitectonics as a method for everything in materials science.

## Data Availability

Data sharing not applicable.
